# (4R*,4aR*,7aS*)-5-Oxo-6-phenyl-4a,5,6,7,7a,8-hexa­hydro-4*H*-furo[2,3-*f*]isoindole-4-carb­oxy­lic acid

**DOI:** 10.1107/S160053681300144X

**Published:** 2013-01-23

**Authors:** Yuriy I. Horak, Roman Z. Lytvyn, Fedor I. Zubkov, Eugeniya V. Nikitina, Yuriy V. Homza, Tadeusz Lis, Vasyl Kinzhybalo, Mykola D. Obushak

**Affiliations:** a Department of Organic Chemistry, Ivan Franko National University of Lviv, Kyryla and Mefodiya 6, Lviv 79005, Ukraine; b Department of Organic Chemistry, Peoples’ Friendship University of Russia, 6 Miklukho-Maklaya St., Moscow 117198, Russian Federation; c Faculty of Chemistry, University of Wrocław, 14 Joliot-Curie St, 50-383 Wrocław, Poland; d Institute of Low Temperature and Structure Research, Okolna 2, 50-422 Wrocław, Poland

## Abstract

The asymmetric unit of the title compound, C_17_H_15_NO_4_, contains two independent mol­ecules with similar geometric parameters. In both mol­ecules, the conformation of the cyclo­hexene ring is half-chair, while the pyrrolidinone ring adopts an envelope conformation with the γ-carbon atom of the α-pyrrolidinone ring as the flap. In the crystal, O—H⋯O hydrogen bonds between the carb­oxylic and carbonyl groups link alternate independent mol­ecules into chains propagating in the *b*-axis direction. The crystal packing also features weak C—H⋯π inter­actions.

## Related literature

For the intra­molecular Diels–Alder reaction of vinyl­furanes, see: Patre *et al.* (2007 ▶). For related solid-phase Diels–Alder reaction with vinyl benzenes, see: Sun *et al.* (2000 ▶). For palladium-catalysed tandem cyclization of allenes with heteroaryl­halides, see: Ohno *et al.* (2005 ▶). For heterolignan derivatives, see: Ramos *et al.* (1999 ▶); Leteurtre *et al.* (1992 ▶) and for their pharmaceutical properties, see: Iwasaki *et al.* (1996 ▶); Ducharme *et al.* (1994 ▶). For a related structure, see: Obushak *et al.* (2011 ▶). For puckering parameters, see: Cremer & Pople (1975 ▶).
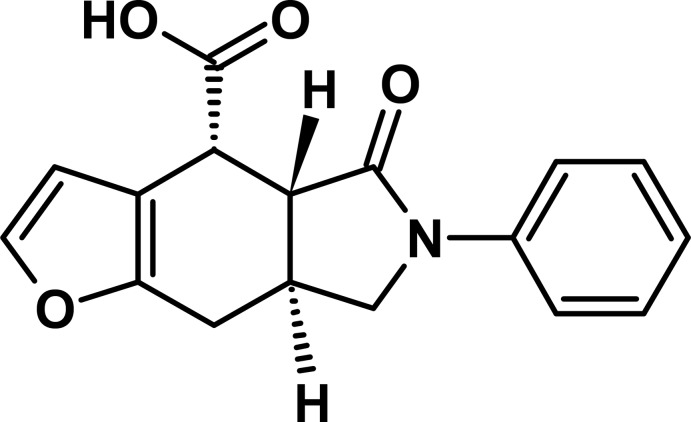


## Experimental

### 

#### Crystal data

C_17_H_15_NO_4_
*M* *_r_* = 297.30
Orthorhombic, 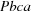

*a* = 12.107 (4) Å
*b* = 16.945 (5) Å
*c* = 27.370 (9) Å
*V* = 5615 (3) Å^3^
*Z* = 16
Mo *K*α radiation
μ = 0.10 mm^−1^
*T* = 120 K
0.64 × 0.42 × 0.28 mm

#### Data collection

Kuma KM-4-CCD diffractometer
Absorption correction: multi-scan (*CrysAlis RED*; Oxford Diffraction, 2006 ▶) *T*_min_ = 0.972, *T*_max_ = 1.000
84648 measured reflections
13130 independent reflections
9304 reflections with *I* > 2σ(*I*)
*R*_int_ = 0.030

#### Refinement

*R*[*F* ^2^ > 2σ(*F* ^2^)] = 0.044
*wR*(*F* ^2^) = 0.127
*S* = 1.03
13130 reflections
399 parameters
H-atom parameters constrained
Δρ_max_ = 0.54 e Å^−3^
Δρ_min_ = −0.21 e Å^−3^

### 

Data collection: *CrysAlis CCD* (Oxford Diffraction, 2006 ▶); cell refinement: *CrysAlis RED* (Oxford Diffraction, 2006 ▶); data reduction: *CrysAlis RED*; program(s) used to solve structure: *SHELXS97* (Sheldrick, 2008 ▶); program(s) used to refine structure: *SHELXL97* (Sheldrick, 2008 ▶); molecular graphics: *DIAMOND* (Brandenburg, 2006 ▶); software used to prepare material for publication: *publCIF* (Westrip, 2010 ▶).

## Supplementary Material

Crystal structure: contains datablock(s) I, global. DOI: 10.1107/S160053681300144X/cv5382sup1.cif

Structure factors: contains datablock(s) I. DOI: 10.1107/S160053681300144X/cv5382Isup2.hkl

Supplementary material file. DOI: 10.1107/S160053681300144X/cv5382Isup3.cdx

Supplementary material file. DOI: 10.1107/S160053681300144X/cv5382Isup4.cml

Additional supplementary materials:  crystallographic information; 3D view; checkCIF report

## Figures and Tables

**Table 1:** Hydrogen-bond geometry (Å, °) *Cg*1 and *Cg*2 are the centroids of the C13*A*–C18*A* and O1*A*–C5*A* rings, respectively.

*D*—H⋯*A*	*D*—H	H⋯*A*	*D*⋯*A*	*D*—H⋯*A*
O3*B*—H3*B*1⋯O4*A*	0.84	1.83	2.6517 (11)	165
O3*A*—H3*A*1⋯O4*B* ^i^	0.84	1.79	2.6329 (10)	178
C8*A*—H8*A*⋯*Cg*1^ii^	1.00	2.50	3.4710 (14)	165
C15*A*—H15*A*⋯*Cg*2^iii^	0.95	2.63	3.5470 (15)	162
C18*A*—H18*B*⋯*Cg*2^iv^	0.99	2.72	3.5492 (14)	141

Symmetry codes: (i) 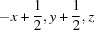; (ii) 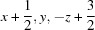; (iii) 
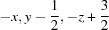
; (iv) 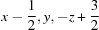.

**Fig. 1.**
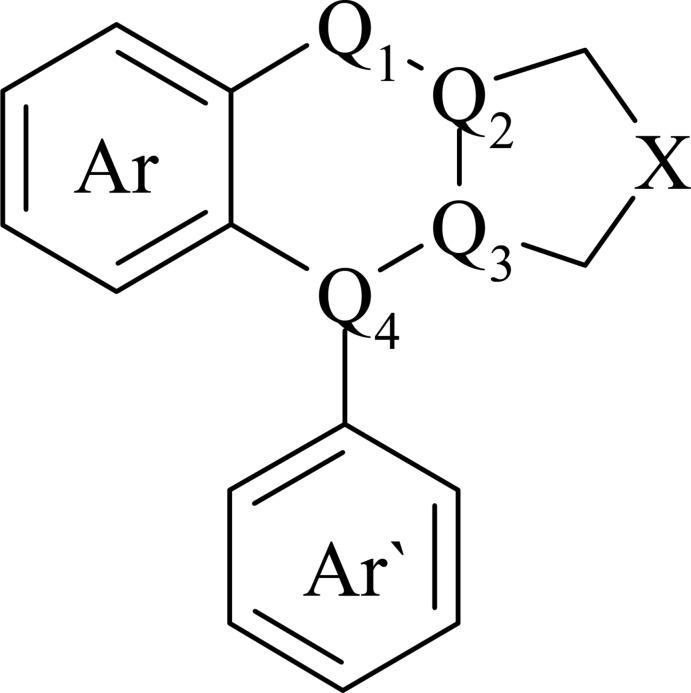
Schematic represenation of heterolignan: X = O, N, S; Q1-4 = C or heteroatom; Ar - Ar' = benzene or heterocycle.

**Fig. 2.**
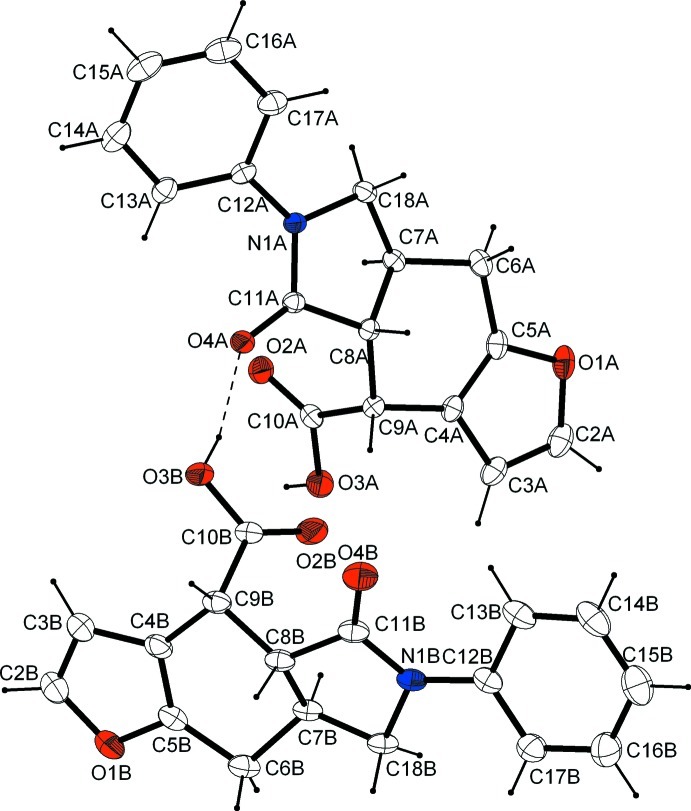
View of two hydrogen-bonded (dashed lines) independent molecules, showing the atom-numbering scheme and 50% probability displacement ellipsoids.

**Fig. 3.**
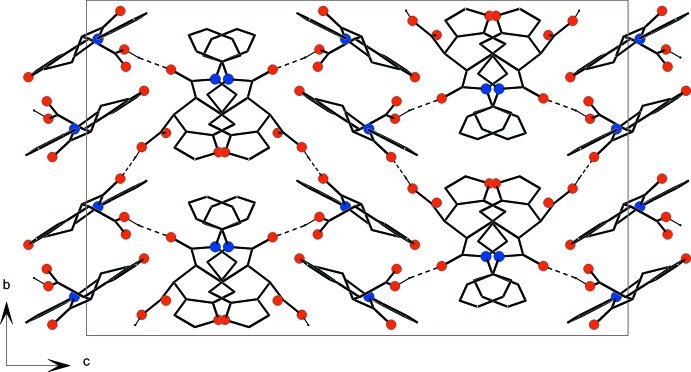
A portion of the crystal packing viewed along the a-axis. Hydrogen atoms not involved in hydrogen bonding were omitted for clarity.

## supplementary crystallographic information

### Comment

Recently, the researchers attention was drawn to such class of compounds as heterolignans (Figure 1) (Ramos *et al.*, 1999). The best known heterolignan is azatoxin, which has antineoplastic activity (Leteurtre *et al.*, 1992). In addition, it should be noted that a number heterolignans show anticancer, antirheumatic and antiasthmatic activity (Iwasaki *et al.*, 1996; Ducharme *et al.*, 1994). There are two important aspects of the synthesis of these compounds. First, as biological activity investigations have shown, the replacement of carbon atoms by heteroatoms in the cycle, or the replacement of benzene fragments by heterocycles, has little effect on biological activity. Second, from the synthetic point of view C–heteroatom bonds are easier accesible than C–C bonds. In addition to this, structural variability and synthetic availability of heterocycles are significantly higher than benzene fragments.

Considering mentioned above, synthesis of lignan analogues or their synthetic precursors, including those with furan cycles, are contemporary tasks. It was found that in the reaction of maleic anhydride and [3-(2-furyl)-2-propenyl]-phenylamine the furane cycle persists and exocyclic double bond reacts. Furoisoindole system with carboxyl group in the six-membered ring is formed. It should be noted that earlier furoisoindole system used to be obtained by the Domino Wittig-Diels-Alder reaction (Patre *et al.*, 2007) and palladium-catalyzed tandem cyclization of allenes with heteroarylhalides (Ohno *et al.*, 2005).

Crystal structure of title compound consists of two independent molecules with very similar geometrical parameters (Figure 2). The five-membered C7—C8—C11—N1—C18 rings of both independent A and B molecules adopt envelope conformation puckered on C7 [puckering parameters (Cremer & Pople, 1975): *q*_2_ = 0.3449 (8) and 0.3525 (9) Å, *φ*_2_ = 283.66 (13) and 287.71 (14)° for A and B molecules, respectively]. The six-membered C4—C5—C6—C7—C8—C9 rings of both independent A and B molecules adopt half-chair conformation (*Q* = 0.5113 (8) and 0.5190 (9) Å, *θ* = 130.33 (9) and 129.98 (10)°, *φ* = 31.02 (12) and 25.34 (13)° for A and B molecules, respectively). There are three chiral carbon atoms (C7, C8 and C9) in the molecule. Two independent molecules are of the same chirality. Since, the compound crystalizes in centrosymmetric space group, it consists of 1:1 ratio mixture of S,*R*,*R*- and *R*,*S*,*S*-isomers.

The structure displays O—H···O hydrogen bonding between acid carboxyl and carbonyl groups, which connects molecules into chains propagating in *b*-axis direction (Figure 3). The crystal packing exhibits weak intermolecular C—H···π interactions.

### Experimental

To a solution of 0.003 mol [3-(2-furyl)-2-propenyl]-phenylamine in benzene 0.003 mol of grinded into a powder maleic anhydride was added. The mixture was boiled until the precipitation of sediment (6–7 h) and 3–4 h thereafter. The precipitate was filtered, washed with benzene and alcohol and recrystalized from EtOH/DMF/H_2_O.

### Refinement

H atoms bonded to O atoms were located in a difference map, but in final refinement cycles O—H distances and C—O—H angles were constrained to 0.84 Å and 109.5°, respectively, with only C—C—O—H torsion angles refined (*U*_iso_(H) = 1.5*U*_eq_(O)). Other H atoms were positioned geometrically and refined using a riding model, with C—H = 0.95–1.00 Å and with *U*_iso_(H) = 1.2*U*_eq_(C).

### Crystal data

**Table:**

C_17_H_15_NO_4_	*F*(000) = 2496
*M**_r_* = 297.30	*D*_x_ = 1.407 Mg m^−^^3^
Orthorhombic, *P**b**c**a*	Mo *K*α radiation, λ = 0.71073 Å
Hall symbol: -P 2ac 2ab	Cell parameters from 39275 reflections
*a* = 12.107 (4) Å	θ = 2.8–36.8°
*b* = 16.945 (5) Å	µ = 0.10 mm^−^^1^
*c* = 27.370 (9) Å	*T* = 120 K
*V* = 5615 (3) Å^3^	Block, brown
*Z* = 16	0.64 × 0.42 × 0.28 mm

### Data collection

**Table:**

Kuma KM-4-CCD diffractometer	13130 independent reflections
Radiation source: fine-focus sealed tube	9304 reflections with *I* > 2σ(*I*)
Graphite monochromator	*R*_int_ = 0.030
ω scan	θ_max_ = 36.9°, θ_min_ = 2.8°
Absorption correction: multi-scan (*CrysAlis RED*; Oxford Diffraction, 2006)	*h* = −20→20
*T*_min_ = 0.972, *T*_max_ = 1.000	*k* = −27→28
84648 measured reflections	*l* = −41→41

### Refinement

**Table:**

Refinement on *F*^2^	Primary atom site location: structure-invariant direct methods
Least-squares matrix: full	Secondary atom site location: difference Fourier map
*R*[*F*^2^ > 2σ(*F*^2^)] = 0.044	Hydrogen site location: inferred from neighbouring sites
*wR*(*F*^2^) = 0.127	H-atom parameters constrained
*S* = 1.03	*w* = 1/[σ^2^(*F*_o_^2^) + (0.08*P*)^2^] where *P* = (*F*_o_^2^ + 2*F*_c_^2^)/3
13130 reflections	(Δ/σ)_max_ = 0.001
399 parameters	Δρ_max_ = 0.54 e Å^−^^3^
0 restraints	Δρ_min_ = −0.21 e Å^−^^3^

### Special details

**Table:**

Geometry. All e.s.d.'s (except the e.s.d. in the dihedral angle between two l.s. planes) are estimated using the full covariance matrix. The cell e.s.d.'s are taken into account individually in the estimation of e.s.d.'s in distances, angles and torsion angles; correlations between e.s.d.'s in cell parameters are only used when they are defined by crystal symmetry. An approximate (isotropic) treatment of cell e.s.d.'s is used for estimating e.s.d.'s involving l.s. planes.
Refinement. Refinement of *F*^2^ against ALL reflections. The weighted *R*-factor *wR* and goodness of fit *S* are based on *F*^2^, conventional *R*-factors *R* are based on *F*, with *F* set to zero for negative *F*^2^. The threshold expression of *F*^2^ > σ(*F*^2^) is used only for calculating *R*-factors(gt) *etc*. and is not relevant to the choice of reflections for refinement. *R*-factors based on *F*^2^ are statistically about twice as large as those based on *F*, and *R*- factors based on ALL data will be even larger.

### Fractional atomic coordinates and isotropic or equivalent isotropic displacement parameters (Å^2^)

**Table:**

	*x*	*y*	*z*	*U*_iso_*/*U*_eq_	
O1A	0.43614 (5)	0.45308 (4)	0.74410 (2)	0.02474 (13)	
C2A	0.48122 (7)	0.47477 (5)	0.70003 (4)	0.02650 (18)	
H2A	0.5417	0.5099	0.6963	0.032*	
C3A	0.42783 (6)	0.43933 (5)	0.66270 (3)	0.02283 (16)	
H3A	0.4434	0.4448	0.6289	0.027*	
C4A	0.34282 (6)	0.39162 (4)	0.68434 (3)	0.01725 (13)	
C5A	0.35163 (6)	0.40237 (4)	0.73337 (3)	0.01896 (14)	
C6A	0.28127 (6)	0.36960 (5)	0.77313 (3)	0.02086 (14)	
H6A1	0.2621	0.4112	0.7971	0.025*	
H6A2	0.3202	0.3265	0.7904	0.025*	
C7A	0.17744 (6)	0.33842 (4)	0.74799 (3)	0.01599 (13)	
H7A	0.1307	0.3844	0.7381	0.019*	
C8A	0.20610 (5)	0.29054 (4)	0.70217 (3)	0.01401 (12)	
H8A	0.2658	0.2530	0.7120	0.017*	
C9A	0.25425 (5)	0.34096 (4)	0.66122 (3)	0.01490 (12)	
H9A	0.2886	0.3059	0.6361	0.018*	
C10A	0.16936 (6)	0.39389 (4)	0.63691 (3)	0.01704 (13)	
O2A	0.07210 (5)	0.39574 (4)	0.64693 (2)	0.02327 (12)	
O3A	0.21636 (5)	0.43830 (4)	0.60227 (2)	0.02464 (13)	
H3A1	0.1690	0.4689	0.5903	0.037*	
C11A	0.10348 (6)	0.24089 (4)	0.69385 (3)	0.01467 (12)	
O4A	0.07488 (4)	0.20698 (3)	0.65625 (2)	0.01945 (11)	
N1A	0.05043 (5)	0.23543 (4)	0.73791 (2)	0.01587 (11)	
C12A	−0.03743 (6)	0.18323 (4)	0.74959 (3)	0.01686 (13)	
C13A	−0.12017 (6)	0.16453 (5)	0.71592 (3)	0.01985 (14)	
H13A	−0.1170	0.1844	0.6835	0.024*	
C14A	−0.20769 (7)	0.11622 (5)	0.73062 (4)	0.02544 (17)	
H14A	−0.2644	0.1037	0.7079	0.031*	
C15A	−0.21328 (7)	0.08613 (5)	0.77770 (4)	0.02926 (19)	
H15A	−0.2735	0.0536	0.7872	0.035*	
C16A	−0.13026 (8)	0.10393 (5)	0.81074 (4)	0.02896 (19)	
H16A	−0.1331	0.0830	0.8429	0.035*	
C17A	−0.04275 (7)	0.15232 (5)	0.79697 (3)	0.02339 (16)	
H17A	0.0137	0.1644	0.8199	0.028*	
C18A	0.10617 (6)	0.28081 (4)	0.77672 (3)	0.01724 (13)	
H18A	0.1520	0.2461	0.7976	0.021*	
H18B	0.0520	0.3090	0.7974	0.021*	
O1B	0.18541 (6)	0.22957 (5)	0.39331 (2)	0.03053 (14)	
C2B	0.07999 (8)	0.21098 (7)	0.40790 (4)	0.0343 (2)	
H2B	0.0149	0.2267	0.3912	0.041*	
C3B	0.08127 (8)	0.16764 (6)	0.44902 (4)	0.03071 (19)	
H3B	0.0190	0.1472	0.4659	0.037*	
C4B	0.19523 (7)	0.15827 (5)	0.46234 (3)	0.02264 (15)	
C5B	0.25439 (7)	0.19673 (5)	0.42746 (3)	0.02408 (16)	
C6B	0.37635 (7)	0.20572 (5)	0.42263 (3)	0.02560 (17)	
H6B1	0.3958	0.2605	0.4135	0.031*	
H6B2	0.4058	0.1693	0.3975	0.031*	
C7B	0.42244 (7)	0.18526 (4)	0.47312 (3)	0.01961 (14)	
H7B	0.4032	0.2288	0.4963	0.024*	
C8B	0.37084 (7)	0.10836 (4)	0.49177 (3)	0.01969 (14)	
H8B	0.3763	0.0694	0.4644	0.024*	
C9B	0.24858 (7)	0.11491 (5)	0.50458 (3)	0.02059 (14)	
H9B	0.2163	0.0607	0.5069	0.025*	
C10B	0.22962 (7)	0.15828 (5)	0.55291 (3)	0.02022 (14)	
O2B	0.29800 (6)	0.19859 (5)	0.57299 (3)	0.03523 (17)	
O3B	0.12847 (5)	0.14681 (4)	0.57001 (2)	0.02688 (14)	
H3B1	0.1208	0.1714	0.5965	0.040*	
C11B	0.45022 (7)	0.08127 (5)	0.53048 (3)	0.02118 (15)	
O4B	0.43207 (6)	0.03220 (4)	0.56273 (3)	0.03127 (15)	
N1B	0.54962 (6)	0.11605 (4)	0.52171 (3)	0.02045 (13)	
C12B	0.65092 (7)	0.09661 (5)	0.54476 (3)	0.02148 (15)	
C13B	0.65372 (9)	0.05946 (5)	0.59053 (3)	0.02803 (18)	
H13B	0.5871	0.0483	0.6075	0.034*	
C14B	0.75534 (10)	0.03908 (6)	0.61076 (4)	0.0346 (2)	
H14B	0.7571	0.0122	0.6412	0.042*	
C15B	0.85333 (10)	0.05686 (6)	0.58777 (4)	0.0375 (2)	
H15B	0.9220	0.0436	0.6024	0.045*	
C16B	0.84995 (9)	0.09450 (7)	0.54291 (4)	0.0361 (2)	
H16B	0.9171	0.1073	0.5268	0.043*	
C17B	0.74999 (8)	0.11391 (6)	0.52103 (4)	0.02780 (17)	
H17B	0.7491	0.1389	0.4900	0.033*	
C18B	0.54527 (7)	0.16734 (5)	0.47787 (3)	0.02155 (15)	
H18C	0.5734	0.1394	0.4486	0.026*	
H18D	0.5886	0.2163	0.4828	0.026*	

### Atomic displacement parameters (Å^2^)

**Table:**

	*U*^11^	*U*^22^	*U*^33^	*U*^12^	*U*^13^	*U*^23^
O1A	0.0215 (3)	0.0201 (3)	0.0327 (3)	−0.0047 (2)	−0.0089 (2)	0.0004 (2)
C2A	0.0198 (3)	0.0208 (3)	0.0388 (5)	−0.0051 (3)	−0.0022 (3)	0.0034 (3)
C3A	0.0179 (3)	0.0196 (3)	0.0310 (5)	−0.0025 (3)	0.0013 (3)	0.0036 (3)
C4A	0.0141 (3)	0.0143 (3)	0.0233 (4)	0.0001 (2)	−0.0011 (2)	0.0008 (2)
C5A	0.0168 (3)	0.0150 (3)	0.0251 (4)	−0.0011 (2)	−0.0048 (3)	−0.0006 (3)
C6A	0.0230 (3)	0.0210 (3)	0.0186 (4)	−0.0020 (3)	−0.0042 (3)	−0.0027 (3)
C7A	0.0180 (3)	0.0158 (3)	0.0141 (3)	0.0005 (2)	−0.0005 (2)	−0.0014 (2)
C8A	0.0136 (2)	0.0134 (3)	0.0150 (3)	0.0009 (2)	0.0001 (2)	−0.0003 (2)
C9A	0.0146 (3)	0.0141 (3)	0.0159 (3)	−0.0001 (2)	0.0007 (2)	−0.0002 (2)
C10A	0.0200 (3)	0.0163 (3)	0.0148 (3)	−0.0008 (2)	−0.0023 (2)	0.0006 (2)
O2A	0.0187 (2)	0.0281 (3)	0.0231 (3)	0.0036 (2)	−0.0021 (2)	0.0048 (2)
O3A	0.0250 (3)	0.0254 (3)	0.0235 (3)	−0.0033 (2)	−0.0026 (2)	0.0101 (2)
C11A	0.0149 (3)	0.0147 (3)	0.0144 (3)	0.0013 (2)	0.0010 (2)	0.0001 (2)
O4A	0.0196 (2)	0.0230 (3)	0.0158 (3)	−0.00351 (19)	0.00158 (19)	−0.0040 (2)
N1A	0.0157 (2)	0.0178 (3)	0.0141 (3)	−0.0016 (2)	0.0019 (2)	−0.0007 (2)
C12A	0.0160 (3)	0.0150 (3)	0.0196 (4)	0.0008 (2)	0.0043 (2)	0.0003 (2)
C13A	0.0174 (3)	0.0181 (3)	0.0241 (4)	0.0002 (2)	0.0025 (3)	−0.0040 (3)
C14A	0.0198 (3)	0.0186 (3)	0.0379 (5)	−0.0023 (3)	0.0053 (3)	−0.0081 (3)
C15A	0.0273 (4)	0.0171 (3)	0.0434 (6)	−0.0043 (3)	0.0129 (4)	−0.0021 (3)
C16A	0.0340 (4)	0.0214 (4)	0.0315 (5)	−0.0022 (3)	0.0113 (4)	0.0057 (3)
C17A	0.0247 (3)	0.0223 (3)	0.0231 (4)	−0.0007 (3)	0.0042 (3)	0.0049 (3)
C18A	0.0194 (3)	0.0192 (3)	0.0131 (3)	−0.0003 (2)	0.0006 (2)	−0.0015 (2)
O1B	0.0337 (3)	0.0394 (4)	0.0184 (3)	0.0012 (3)	−0.0039 (2)	0.0016 (3)
C2B	0.0307 (4)	0.0488 (6)	0.0234 (5)	0.0004 (4)	−0.0054 (3)	−0.0050 (4)
C3B	0.0286 (4)	0.0421 (5)	0.0214 (4)	−0.0035 (4)	−0.0015 (3)	−0.0063 (4)
C4B	0.0276 (4)	0.0249 (4)	0.0154 (4)	−0.0011 (3)	0.0012 (3)	−0.0051 (3)
C5B	0.0304 (4)	0.0263 (4)	0.0155 (4)	0.0019 (3)	−0.0002 (3)	−0.0016 (3)
C6B	0.0307 (4)	0.0283 (4)	0.0178 (4)	0.0013 (3)	0.0048 (3)	0.0043 (3)
C7B	0.0261 (3)	0.0170 (3)	0.0158 (4)	0.0007 (3)	0.0049 (3)	0.0016 (3)
C8B	0.0274 (3)	0.0150 (3)	0.0167 (4)	−0.0004 (3)	0.0052 (3)	−0.0014 (2)
C9B	0.0261 (3)	0.0189 (3)	0.0168 (4)	−0.0027 (3)	0.0039 (3)	−0.0027 (3)
C10B	0.0239 (3)	0.0211 (3)	0.0157 (4)	−0.0003 (3)	0.0039 (3)	0.0000 (3)
O2B	0.0313 (3)	0.0474 (4)	0.0270 (4)	−0.0132 (3)	0.0095 (3)	−0.0181 (3)
O3B	0.0231 (3)	0.0381 (4)	0.0194 (3)	−0.0034 (2)	0.0054 (2)	−0.0073 (3)
C11B	0.0292 (4)	0.0155 (3)	0.0188 (4)	0.0000 (3)	0.0057 (3)	0.0010 (3)
O4B	0.0378 (4)	0.0270 (3)	0.0291 (4)	−0.0052 (3)	0.0039 (3)	0.0128 (3)
N1B	0.0272 (3)	0.0176 (3)	0.0166 (3)	0.0006 (2)	0.0043 (2)	0.0030 (2)
C12B	0.0303 (4)	0.0166 (3)	0.0176 (4)	0.0010 (3)	−0.0001 (3)	−0.0007 (3)
C13B	0.0419 (5)	0.0233 (4)	0.0189 (4)	−0.0009 (3)	−0.0013 (3)	0.0017 (3)
C14B	0.0524 (6)	0.0271 (4)	0.0244 (5)	0.0005 (4)	−0.0105 (4)	0.0045 (3)
C15B	0.0417 (5)	0.0322 (5)	0.0387 (6)	0.0052 (4)	−0.0128 (4)	0.0026 (4)
C16B	0.0300 (4)	0.0414 (5)	0.0367 (6)	0.0021 (4)	−0.0035 (4)	0.0048 (4)
C17B	0.0291 (4)	0.0304 (4)	0.0239 (4)	0.0010 (3)	−0.0002 (3)	0.0044 (3)
C18B	0.0270 (3)	0.0209 (3)	0.0168 (4)	0.0016 (3)	0.0061 (3)	0.0047 (3)

### Geometric parameters (Å, º)

**Table:**

O1A—C5A	1.3681 (9)	O1B—C5B	1.3715 (11)
O1A—C2A	1.3740 (12)	O1B—C2B	1.3739 (13)
C2A—C3A	1.3500 (13)	C2B—C3B	1.3440 (16)
C2A—H2A	0.9500	C2B—H2B	0.9500
C3A—C4A	1.4364 (11)	C3B—C4B	1.4359 (13)
C3A—H3A	0.9500	C3B—H3B	0.9500
C4A—C5A	1.3585 (13)	C4B—C5B	1.3598 (13)
C4A—C9A	1.5124 (10)	C4B—C9B	1.5144 (13)
C5A—C6A	1.4893 (12)	C5B—C6B	1.4902 (14)
C6A—C7A	1.5273 (11)	C6B—C7B	1.5301 (13)
C6A—H6A1	0.9900	C6B—H6B1	0.9900
C6A—H6A2	0.9900	C6B—H6B2	0.9900
C7A—C18A	1.5218 (11)	C7B—C18B	1.5234 (12)
C7A—C8A	1.5335 (11)	C7B—C8B	1.5326 (11)
C7A—H7A	1.0000	C7B—H7B	1.0000
C8A—C11A	1.5175 (10)	C8B—C11B	1.5024 (13)
C8A—C9A	1.5251 (10)	C8B—C9B	1.5251 (12)
C8A—H8A	1.0000	C8B—H8B	1.0000
C9A—C10A	1.5178 (10)	C9B—C10B	1.5307 (12)
C9A—H9A	1.0000	C9B—H9B	1.0000
C10A—O2A	1.2094 (10)	C10B—O2B	1.2057 (11)
C10A—O3A	1.3375 (10)	C10B—O3B	1.3254 (10)
O3A—H3A1	0.8400	O3B—H3B1	0.8400
C11A—O4A	1.2286 (9)	C11B—O4B	1.2324 (10)
C11A—N1A	1.3696 (10)	C11B—N1B	1.3613 (11)
N1A—C12A	1.4199 (10)	N1B—C12B	1.4181 (12)
N1A—C18A	1.4747 (10)	N1B—C18B	1.4825 (11)
C12A—C13A	1.3976 (12)	C12B—C17B	1.3953 (13)
C12A—C17A	1.4000 (12)	C12B—C13B	1.4024 (13)
C13A—C14A	1.3981 (11)	C13B—C14B	1.3926 (15)
C13A—H13A	0.9500	C13B—H13B	0.9500
C14A—C15A	1.3875 (15)	C14B—C15B	1.3763 (17)
C14A—H14A	0.9500	C14B—H14B	0.9500
C15A—C16A	1.3852 (15)	C15B—C16B	1.3843 (16)
C15A—H15A	0.9500	C15B—H15B	0.9500
C16A—C17A	1.3916 (12)	C16B—C17B	1.3898 (14)
C16A—H16A	0.9500	C16B—H16B	0.9500
C17A—H17A	0.9500	C17B—H17B	0.9500
C18A—H18A	0.9900	C18B—H18C	0.9900
C18A—H18B	0.9900	C18B—H18D	0.9900
			
C5A—O1A—C2A	106.06 (7)	C5B—O1B—C2B	105.94 (8)
C3A—C2A—O1A	110.80 (7)	C3B—C2B—O1B	110.97 (9)
C3A—C2A—H2A	124.6	C3B—C2B—H2B	124.5
O1A—C2A—H2A	124.6	O1B—C2B—H2B	124.5
C2A—C3A—C4A	106.34 (8)	C2B—C3B—C4B	106.51 (9)
C2A—C3A—H3A	126.8	C2B—C3B—H3B	126.7
C4A—C3A—H3A	126.8	C4B—C3B—H3B	126.7
C5A—C4A—C3A	106.00 (7)	C5B—C4B—C3B	105.95 (8)
C5A—C4A—C9A	123.02 (7)	C5B—C4B—C9B	122.94 (8)
C3A—C4A—C9A	130.92 (8)	C3B—C4B—C9B	131.10 (8)
C4A—C5A—O1A	110.79 (7)	C4B—C5B—O1B	110.62 (8)
C4A—C5A—C6A	128.83 (7)	C4B—C5B—C6B	129.29 (8)
O1A—C5A—C6A	120.34 (7)	O1B—C5B—C6B	120.09 (8)
C5A—C6A—C7A	105.69 (7)	C5B—C6B—C7B	104.95 (7)
C5A—C6A—H6A1	110.6	C5B—C6B—H6B1	110.8
C7A—C6A—H6A1	110.6	C7B—C6B—H6B1	110.8
C5A—C6A—H6A2	110.6	C5B—C6B—H6B2	110.8
C7A—C6A—H6A2	110.6	C7B—C6B—H6B2	110.8
H6A1—C6A—H6A2	108.7	H6B1—C6B—H6B2	108.8
C18A—C7A—C6A	117.12 (7)	C18B—C7B—C6B	118.57 (7)
C18A—C7A—C8A	102.21 (6)	C18B—C7B—C8B	101.53 (6)
C6A—C7A—C8A	111.43 (6)	C6B—C7B—C8B	110.17 (7)
C18A—C7A—H7A	108.6	C18B—C7B—H7B	108.7
C6A—C7A—H7A	108.6	C6B—C7B—H7B	108.7
C8A—C7A—H7A	108.6	C8B—C7B—H7B	108.7
C11A—C8A—C9A	120.88 (6)	C11B—C8B—C9B	118.75 (7)
C11A—C8A—C7A	103.34 (6)	C11B—C8B—C7B	103.53 (7)
C9A—C8A—C7A	113.02 (6)	C9B—C8B—C7B	114.22 (7)
C11A—C8A—H8A	106.2	C11B—C8B—H8B	106.5
C9A—C8A—H8A	106.2	C9B—C8B—H8B	106.5
C7A—C8A—H8A	106.2	C7B—C8B—H8B	106.5
C4A—C9A—C10A	109.15 (6)	C4B—C9B—C8B	105.89 (7)
C4A—C9A—C8A	106.36 (6)	C4B—C9B—C10B	111.28 (7)
C10A—C9A—C8A	113.23 (6)	C8B—C9B—C10B	112.28 (7)
C4A—C9A—H9A	109.3	C4B—C9B—H9B	109.1
C10A—C9A—H9A	109.3	C8B—C9B—H9B	109.1
C8A—C9A—H9A	109.3	C10B—C9B—H9B	109.1
O2A—C10A—O3A	124.08 (7)	O2B—C10B—O3B	123.81 (8)
O2A—C10A—C9A	125.12 (7)	O2B—C10B—C9B	124.26 (7)
O3A—C10A—C9A	110.80 (7)	O3B—C10B—C9B	111.93 (7)
C10A—O3A—H3A1	109.5	C10B—O3B—H3B1	109.5
O4A—C11A—N1A	125.02 (7)	O4B—C11B—N1B	125.18 (8)
O4A—C11A—C8A	128.01 (6)	O4B—C11B—C8B	126.67 (8)
N1A—C11A—C8A	106.82 (6)	N1B—C11B—C8B	107.98 (7)
C11A—N1A—C12A	126.28 (6)	C11B—N1B—C12B	125.83 (7)
C11A—N1A—C18A	112.60 (6)	C11B—N1B—C18B	111.42 (7)
C12A—N1A—C18A	120.36 (6)	C12B—N1B—C18B	121.81 (7)
C13A—C12A—C17A	119.54 (7)	C17B—C12B—C13B	119.31 (8)
C13A—C12A—N1A	121.99 (7)	C17B—C12B—N1B	119.17 (8)
C17A—C12A—N1A	118.43 (7)	C13B—C12B—N1B	121.51 (8)
C12A—C13A—C14A	119.09 (8)	C14B—C13B—C12B	119.20 (9)
C12A—C13A—H13A	120.5	C14B—C13B—H13B	120.4
C14A—C13A—H13A	120.5	C12B—C13B—H13B	120.4
C15A—C14A—C13A	121.29 (8)	C15B—C14B—C13B	121.70 (9)
C15A—C14A—H14A	119.4	C15B—C14B—H14B	119.2
C13A—C14A—H14A	119.4	C13B—C14B—H14B	119.2
C16A—C15A—C14A	119.40 (8)	C14B—C15B—C16B	118.75 (10)
C16A—C15A—H15A	120.3	C14B—C15B—H15B	120.6
C14A—C15A—H15A	120.3	C16B—C15B—H15B	120.6
C15A—C16A—C17A	120.26 (9)	C15B—C16B—C17B	121.13 (10)
C15A—C16A—H16A	119.9	C15B—C16B—H16B	119.4
C17A—C16A—H16A	119.9	C17B—C16B—H16B	119.4
C16A—C17A—C12A	120.41 (9)	C16B—C17B—C12B	119.88 (9)
C16A—C17A—H17A	119.8	C16B—C17B—H17B	120.1
C12A—C17A—H17A	119.8	C12B—C17B—H17B	120.1
N1A—C18A—C7A	102.82 (6)	N1B—C18B—C7B	102.75 (6)
N1A—C18A—H18A	111.2	N1B—C18B—H18C	111.2
C7A—C18A—H18A	111.2	C7B—C18B—H18C	111.2
N1A—C18A—H18B	111.2	N1B—C18B—H18D	111.2
C7A—C18A—H18B	111.2	C7B—C18B—H18D	111.2
H18A—C18A—H18B	109.1	H18C—C18B—H18D	109.1
			
C5A—O1A—C2A—C3A	−0.07 (9)	C5B—O1B—C2B—C3B	−0.68 (11)
O1A—C2A—C3A—C4A	−0.06 (9)	O1B—C2B—C3B—C4B	0.72 (12)
C2A—C3A—C4A—C5A	0.16 (9)	C2B—C3B—C4B—C5B	−0.48 (11)
C2A—C3A—C4A—C9A	177.41 (7)	C2B—C3B—C4B—C9B	−179.30 (9)
C3A—C4A—C5A—O1A	−0.22 (8)	C3B—C4B—C5B—O1B	0.07 (10)
C9A—C4A—C5A—O1A	−177.73 (6)	C9B—C4B—C5B—O1B	179.02 (7)
C3A—C4A—C5A—C6A	177.26 (7)	C3B—C4B—C5B—C6B	−179.45 (9)
C9A—C4A—C5A—C6A	−0.25 (12)	C9B—C4B—C5B—C6B	−0.51 (14)
C2A—O1A—C5A—C4A	0.18 (9)	C2B—O1B—C5B—C4B	0.35 (10)
C2A—O1A—C5A—C6A	−177.54 (7)	C2B—O1B—C5B—C6B	179.93 (8)
C4A—C5A—C6A—C7A	−14.66 (11)	C4B—C5B—C6B—C7B	−17.32 (12)
O1A—C5A—C6A—C7A	162.62 (6)	O1B—C5B—C6B—C7B	163.19 (7)
C5A—C6A—C7A—C18A	162.60 (6)	C5B—C6B—C7B—C18B	164.07 (7)
C5A—C6A—C7A—C8A	45.46 (8)	C5B—C6B—C7B—C8B	47.79 (9)
C18A—C7A—C8A—C11A	33.72 (7)	C18B—C7B—C8B—C11B	33.71 (8)
C6A—C7A—C8A—C11A	159.58 (6)	C6B—C7B—C8B—C11B	160.23 (6)
C18A—C7A—C8A—C9A	166.08 (6)	C18B—C7B—C8B—C9B	164.30 (7)
C6A—C7A—C8A—C9A	−68.05 (8)	C6B—C7B—C8B—C9B	−69.18 (9)
C5A—C4A—C9A—C10A	106.75 (8)	C5B—C4B—C9B—C8B	−12.99 (10)
C3A—C4A—C9A—C10A	−70.09 (10)	C3B—C4B—C9B—C8B	165.66 (9)
C5A—C4A—C9A—C8A	−15.74 (9)	C5B—C4B—C9B—C10B	109.28 (9)
C3A—C4A—C9A—C8A	167.42 (7)	C3B—C4B—C9B—C10B	−72.07 (11)
C11A—C8A—C9A—C4A	170.99 (6)	C11B—C8B—C9B—C4B	169.01 (7)
C7A—C8A—C9A—C4A	47.89 (7)	C7B—C8B—C9B—C4B	46.37 (9)
C11A—C8A—C9A—C10A	51.12 (9)	C11B—C8B—C9B—C10B	47.39 (9)
C7A—C8A—C9A—C10A	−71.98 (8)	C7B—C8B—C9B—C10B	−75.25 (9)
C4A—C9A—C10A—O2A	−120.49 (8)	C4B—C9B—C10B—O2B	−102.12 (10)
C8A—C9A—C10A—O2A	−2.23 (11)	C8B—C9B—C10B—O2B	16.37 (12)
C4A—C9A—C10A—O3A	60.04 (8)	C4B—C9B—C10B—O3B	77.38 (9)
C8A—C9A—C10A—O3A	178.30 (6)	C8B—C9B—C10B—O3B	−164.12 (7)
C9A—C8A—C11A—O4A	33.83 (11)	C9B—C8B—C11B—O4B	35.02 (12)
C7A—C8A—C11A—O4A	161.42 (7)	C7B—C8B—C11B—O4B	162.85 (8)
C9A—C8A—C11A—N1A	−150.61 (6)	C9B—C8B—C11B—N1B	−149.49 (7)
C7A—C8A—C11A—N1A	−23.01 (7)	C7B—C8B—C11B—N1B	−21.66 (8)
O4A—C11A—N1A—C12A	8.13 (12)	O4B—C11B—N1B—C12B	6.42 (14)
C8A—C11A—N1A—C12A	−167.60 (6)	C8B—C11B—N1B—C12B	−169.16 (7)
O4A—C11A—N1A—C18A	178.15 (7)	O4B—C11B—N1B—C18B	175.43 (8)
C8A—C11A—N1A—C18A	2.42 (8)	C8B—C11B—N1B—C18B	−0.14 (9)
C11A—N1A—C12A—C13A	−37.46 (11)	C11B—N1B—C12B—C17B	156.19 (8)
C18A—N1A—C12A—C13A	153.23 (7)	C18B—N1B—C12B—C17B	−11.76 (12)
C11A—N1A—C12A—C17A	145.01 (8)	C11B—N1B—C12B—C13B	−22.92 (12)
C18A—N1A—C12A—C17A	−24.30 (10)	C18B—N1B—C12B—C13B	169.13 (8)
C17A—C12A—C13A—C14A	1.04 (11)	C17B—C12B—C13B—C14B	−1.42 (13)
N1A—C12A—C13A—C14A	−176.46 (7)	N1B—C12B—C13B—C14B	177.70 (8)
C12A—C13A—C14A—C15A	−0.55 (12)	C12B—C13B—C14B—C15B	2.25 (15)
C13A—C14A—C15A—C16A	−0.35 (13)	C13B—C14B—C15B—C16B	−1.38 (16)
C14A—C15A—C16A—C17A	0.76 (13)	C14B—C15B—C16B—C17B	−0.32 (17)
C15A—C16A—C17A—C12A	−0.26 (13)	C15B—C16B—C17B—C12B	1.10 (16)
C13A—C12A—C17A—C16A	−0.65 (12)	C13B—C12B—C17B—C16B	−0.21 (14)
N1A—C12A—C17A—C16A	176.94 (7)	N1B—C12B—C17B—C16B	−179.34 (9)
C11A—N1A—C18A—C7A	19.30 (8)	C11B—N1B—C18B—C7B	21.95 (9)
C12A—N1A—C18A—C7A	−170.02 (6)	C12B—N1B—C18B—C7B	−168.52 (7)
C6A—C7A—C18A—N1A	−154.05 (6)	C6B—C7B—C18B—N1B	−154.21 (7)
C8A—C7A—C18A—N1A	−32.00 (7)	C8B—C7B—C18B—N1B	−33.41 (8)

### Hydrogen-bond geometry (Å, º)

Cg1 and Cg2 are the centroids of the C13A–C18A and O1A–C5A rings, respectively.

**Table:**

*D*—H···*A*	*D*—H	H···*A*	*D*···*A*	*D*—H···*A*
O3*B*—H3*B*1···O4*A*	0.84	1.83	2.6517 (11)	165
O3*A*—H3*A*1···O4*B*^i^	0.84	1.79	2.6329 (10)	178
C8*A*—H8*A*···*Cg*1^ii^	1.00	2.50	3.4710 (14)	165
C15*A*—H15*A*···*Cg*2^iii^	0.95	2.63	3.5470 (15)	162
C18*A*—H18*B*···*Cg*2^iv^	0.99	2.72	3.5492 (14)	141

Symmetry codes: (i) −*x*+1/2, *y*+1/2, *z*; (ii) *x*+1/2, *y*, −*z*+3/2; (iii) −*x*, *y*−1/2, −*z*+3/2; (iv) *x*−1/2, *y*, −*z*+3/2.
